# Changes in the Phase Composition of Calcium Aluminoferrites Based on the Synthesis Condition and Al_2_O_3_/Fe_2_O_3_ Molar Ratio

**DOI:** 10.3390/ma16124234

**Published:** 2023-06-07

**Authors:** Michał Pyzalski, Tomasz Brylewski, Agnieszka Sujak, Karol Durczak

**Affiliations:** 1 Faculty of Materials Science and Ceramics, AGH University of Science and Technology, Al. Mickiewicza 30, 30-059 Kraków, Poland; brylew@agh.edu.pl; 2 Department of Biosystems Engineering, Faculty of Environmental and Mechanical Engineering, Poznań University of Life Sciences, Wojska Polskiego 50 Street, 60-627 Poznań, Poland; agnieszka.sujak@up.poznan.pl (A.S.); karol.durczak@up.poznan.pl (K.D.)

**Keywords:** calcium aluminoferrites, synthesis condition, solid solutions, X-ray diffraction

## Abstract

The presented work concerns the study of the changes in the phase composition of calcium aluminoferrites which depend on the synthesis conditions and the selection of the Al_2_O_3_/Fe_2_O_3_ molar ratio (A/F). The A/F molar ratio extends beyond the limiting composition of C_6_A_2_F (6CaO·2Al_2_O_3_·Fe_2_O) towards phases richer in Al_2_O_3_. An increase in the A/F ratio above unity favours the formation of other crystalline phases such as C_12_A_7_ and C_3_A, in addition to calcium aluminoferrite. Slow cooling of melts characterised by an A/F ratio below 0.58, results in the formation of a single calcium aluminoferrite phase. Above this ratio, the presence of varying contents of C_12_A_7_ and C_3_A phases was found. The process of rapid cooling of the melts with an A/F molar ratio approaching the value of four favours the formation of a single phase with variable chemical composition. Generally, an increase in the A/F ratio above the value of four generates the formation of a calcium aluminoferrite amorphous phase. The rapidly cooled samples with compositions of C_22.19_A_10.94_F and C_14.61_A_6.29_F were fully amorphous. Additionally, this study shows that as the A/F molar ratio of the melts decreases, the elemental cell volume of the calcium aluminoferrites decreases.

## 1. Introduction

**Figure 1:**
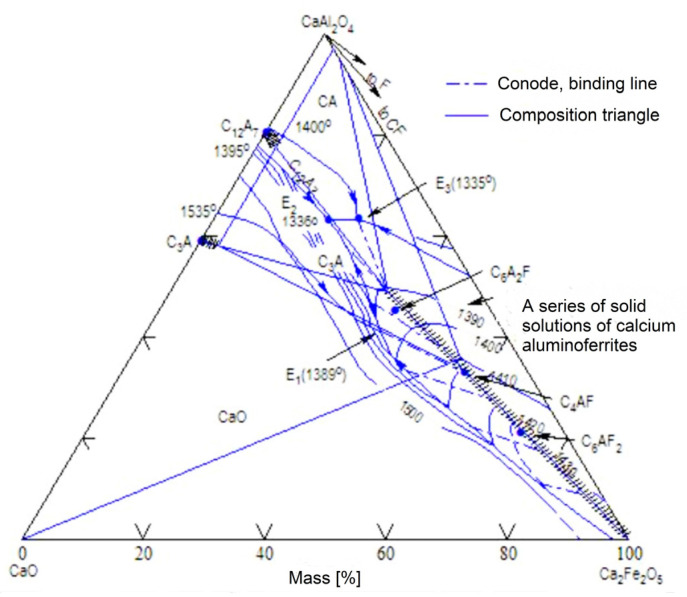
Phase diagram of the CaO-2CaO·Fe_2_O_3_-CaO·Al_2_O_3_ system [2].

In the C-A-F (CaO-Al_2_O_3_-Fe_2_O_3_) ternary system (Figure 1), a series of solid solutions are present on the line starting at the point of C_2_F composition (2CaO·Fe_2_O_3_), and along the line joining the hypothetical C_2_A (2CaO·Al_2_O_3_) up to a composition expressed by an A/F(Al_2_O_3_/Fe_2_O_3_) ratio of 0.69 [1]. Previous experiments showed that the line between the hypothetical C_2_A and C_2_F phases can be divided into three ranges [2]. The first is the range of occurrence of solid solutions of calcium aluminoferrites in the area between C_2_F and C_4_AF. The second range is the area of occurrence of solid solutions between C_4_AF and the compound defined as C_6_A_2_F. Within the above area, it is also possible to delineate a compositional range between the compound C_6_AF_2_ and C_6_A_2_F. 

This phenomenon is associated with the occurrence of calcium aluminoferrites in Portland clinker, and it is also the subject of numerous research works [3]. The third, least understood, compositional range is that of the phases occurring on the line between C_6_A_2_F and the hypothetical C_2_A.

Studies [4,5] show that solid solutions located between C_2_F and the hypothetical C_2_A are beyond the limiting range of C_4_AF. This can have a significant impact on the properties of Portland clinker and its hydration activity. According to Bojkova [6], calcium aluminoferrites in industrial Portland clinker should be identified with the C_6_A_2_F phase rather than with C_4_AF, which may be explained by the use of imprecise calculation techniques for clinker phase composition determination methods.

The region in the C-A-F configuration between the hypothetical C_2_A and the C_4_AF phase was previously investigated by Swayz [7], Newkirk [8], and Smith [9]. The results of the aforementioned work confirmed that C_4_AF is not an extreme member of a range of calcium aluminoferrite solid solutions, while the C_6_A_2_F phase was considered a limiting compound that is still a solid solution [10].

The C_4_AF phase, which was most extensively reported in the literature, occurs as one of the elements of a series of Ca_2_Fe_2−x_Al_x_O_5_ solid solutions in which x takes on values ranging from 0.66 to 1.38. The crystal structure of this compound was classified into the spatial ‘Icmm’ group. The structure of the C_4_AF phase is similar to that of C_2_F and consists of perovskite-like layers. The average distance between atoms in C_4_AF polyhedra, consisting of tetrahedra and octahedra, is slightly smaller than in the C_2_F phase. The polyhedra forming the C_4_AF structure become distorted as a result of the failure to maintain the correct proportions in the arrangement of the polyhedral elements [10].

The calcium ion is located in a deformed polyhedra containing seven oxygen ions. The average distance between the calcium and oxygen ions is assumed to be 2.461 Å. Consequently, the polyhedra in C_2_F and C_4_AF should be characterised by high chemical bond anisotropy, which, in combination with the electric field gradient of the Fe^3+^ ion, favours the generation of stresses within the crystal structure [11]. This results in an increase in the activity level of these compounds and the dynamics of solid-phase reactions including hydration processes. In the structure of calcium aluminoferrites, aluminium ions substitute iron ions predominantly in the tetrahedral positions up to x = 0.33, i.e., in the anionic lattice, followed by substitutions taking place evenly in both co-ordinations and involving both tetrahedral and octahedral positions.

**Figure 2:**
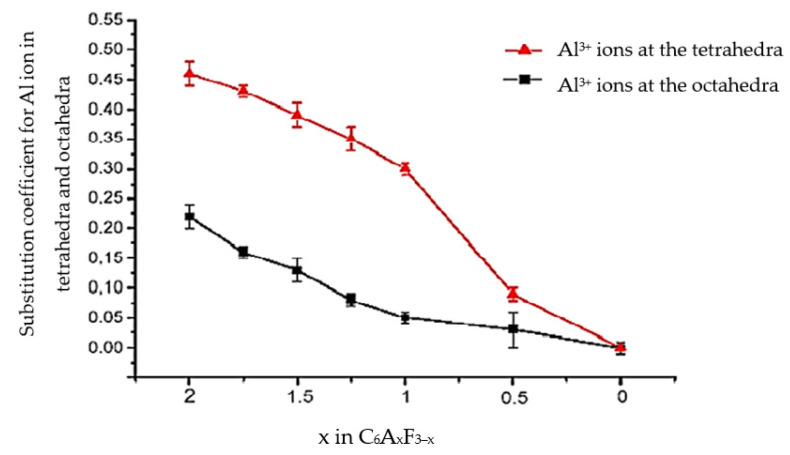
Relationship between the value of the Al substitution factor for Fe in the tetrahedral and octahedral positions of calcium aluminoferrites according to [2].

A smaller ionic radius of Al^3+^ (0.57 Å) compared to the Fe^3+^ ion (0.67 Å) favours the accelerated migration of Al^3+^ ions from the tetrahedral to the octahedral position [12]. Figure 2 shows the results of study [2], describing the changes in the A/F ratio as a result of the substitution of Fe^3+^ ions with Al^3+^ ions in the tetrahedral and octahedral positions of calcium aluminoferrites.

The clinkerisation process involves the phenomenon of heterovalent isomorphic substitutions in the elemental cell of calcium aluminoferrites, which de facto disrupt the electrostatic balance and micro-symmetry of the structure. The accumulation of structural defects may favour the formation of calcium aluminoferrites in amorphous form.

Studies on the determination of elemental cell parameters of a series of C_2_F solid solutions and a hypothetical ‘C_2_A’ in the C-A-F system were carried out by Newkirk [8], Thwaite [8], and Smith [9]. The results of these studies, together with supplemented data from the ICCD PDF-2 database, are summarized in Table 1.

Calcium aluminoferrite phases are one of the main compounds included in several types of bonding materials, e.g., Portland cement and aluminous cement [13,14].

The problem of the varying properties of calcium aluminoferrites formed by sintering in the presence of a small amount of liquid phase and crystallising from the melt, as well as the influence of the melt cooling conditions on the type and properties of these phases, has been the subject of very few works. One of the first researchers to determine the composition of the aluminoferrite phase described by the C_4_AF formula was Brownmiller [3]. The found mass ratio of Al_2_O_3_/Fe_2_O_3_ in the examined phase was 0.64, and several solid solutions between the C_2_F and C_4_AF compositions were also indicated. The CaO-Al_2_O_3_-Fe_2_O_3_ system was also studied by Yamauchi [11] and Swayz [7]. These authors showed that the solid solutions between C_2_F and the hypothetical C_2_A are beyond the ‘extreme’ range of C_4_AF, which has a significant impact on the properties of the Portland clinker and, subsequently, on the properties of the resulting cements. Comparatively, Newkirk and Thwaite [8], noted that C_4_AF is not an ‘extreme’ solid solution of a range of calcium aluminoferrites. According to these authors, the limiting solid solution enriched in aluminium is C_6_A_2_F. Considering these facts, further research directed at determining the maximum content of aluminium ions in solid solutions that correspond to the extreme composition of C_6_A_2_F is justified.

The present study aims to determine both the influence of the A/F molar ratio beyond the limiting composition of C_6_A_2_F towards Al_2_O_3_-richer phases and the effect of the synthesis conditions on the phase composition of calcium aluminoferrites.

In this paper, we present the synthesis of aluminoferrites sequentially by showing the varying starting compositions of the mixtures, the melting and cooling conditions (slow or rapid cooling), and the final result in the analysis of the phases formed depending on the A/F ratio and the cooling method.

In studies planned for the near future, pro-ecological solutions for the use of chemically and structurally modified calcium aluminoferrites in the production of modern binding materials will be proposed.

## 2. Materials and Methods

### 2.1. Component Materials for Synthesis

Calcium carbonate, aluminium oxide, and iron (III) oxide with a purity of p.a. (POCh S.A. Gliwice, Poland) were used to obtain calcium aluminoferrites. The composition of the raw material mixtures was chosen to correspond to the nominal compositions for the following chemical combinations: C_2_F, C_22_AF_10_, C_12_AF_5_, C_8_AF_3_, C_6_AF_2_, C_4_AF, C_6_A_2_F, C_10_A_4_F, and C_22_A_10_F. The choice of the individual oxides and the molar and mass ratio A/F of the calcium aluminoferrites are given in Table 2.

The A/F molar ratio of the starting compositions of the mixtures varied in the range of 0.1 to 10.0, while the CaO/(Al_2_O_3_ + Fe_2_O_3_) constant molar ratio of two was kept.

### 2.2. Methods of Synthesis

**Figure 3:**
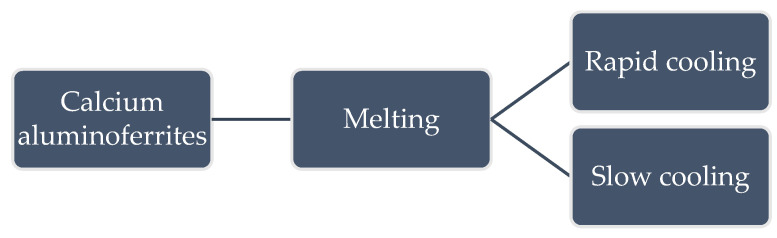
Scheme for the preparation of calcium aluminoferrites.

Calcium aluminoferrites were obtained by melting the raw material stock. The formation of the structure and microstructure of the samples was carried out in two ways: the first involved slow cooling, while the second involved rapid cooling of the produced melts, according to the scheme shown in Figure 3.

The process of high-temperature thermal treatment of samples with a diameter of 50 mm and a height of 80 mm placed in platinum evaporators was carried out in a superkanthal furnace. The samples were prepared from a set of mixtures moistened with water at 7–8%, which were densified and then dried at 110 °C. The melting process was carried out by heating the samples at a temperature 20 °C higher than the characteristic temperature taken from the literature data [8]. The resulting melts were held at peak temperature for 2 h. The tests involved the preparation of two series of samples: slowly and rapidly cooled. Rapid cooling involved immediately removing the evaporators with the samples from the furnace and placing them in a water bath at 10 °C. Slow cooling comprised leaving the melted material in the furnace cooled at an average rate of about 2 °C/min to 1000 °C and then at a rate of about 10 °C/min to 400 °C. Once this temperature was reached, the samples were removed from the oven and cooled at room temperature. In the next step, the samples were initially crushed in an agate mortar (Conbest, Cracow, Poland) and then ground in a vibratory mill to a grain size corresponding to a 0.01 mm sieve mesh (Frisch GmbH & Co KG, Munich, Germany). Identical grinding conditions were applied for all the preparations tested.

The content of CaO, Fe_2_O_3_, and Al_2_O_3_ in the obtained samples was determined in accordance with PN-EN 196-2:2006 [15]. The results of the actual molar ratios of the oxides in the individual variants are summarised in Table 3.

It can be seen that the actual molar compositions of the calcium aluminoferrites obtained are in approximate agreement with the nominal compositions, justifying a study of their phase composition.

### 2.3. Methods of Determination of the Sintering, Melting, and Flow Temperatures of the Samples

The homogenised samples were examined in a high-temperature heating microscope (Hesse Instrumente, Osterode am Harz, Germany). The samples used for this study were 1 g cube-shaped samples, which were obtained by powder moulding in a punch press (Metimex, Pyskowice, Poland). The thermal processing of the samples was carried out by a linear temperature increase at a rate of 5 °C/min. The heating microscope was equipped with specialised software allowing quantitative measurements of the outline of the surface area (in %) and the changes in the shape of the top line of the moulded sample cube. This enabled the temperatures of the onset of sintering, melting, and flowing of the samples to be determined with high precision.

Computer analysis of the heat treatment process allows curves to be plotted with “x” and “y” coordinates, representing, respectively, the current temperature of the sample and changes in the outline of the pellet’s surface area. The computer software allowed for directly obtaining information on the amount of the separated liquid phase of the examined sample based on changes in the outline of the analysed surface area. It was assumed that at the sample’s melting temperature, the sample would melt completely, which corresponds to a 100% liquid phase separation.

**Figure 4:**
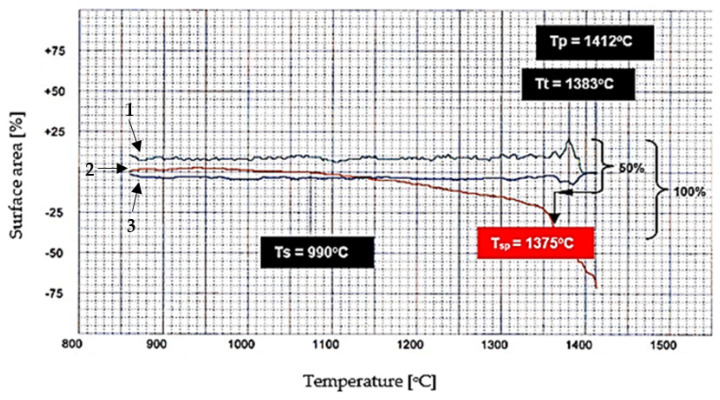
An example of the determination of the characteristic heat treatment temperature C_6_AF_2_, where T_s_—temperature of the start of sintering (experimental); T_sp_—temperature of the start of sintering (calculated); T_t_—temperature of melting; T_p_—temperature of flow. 1—sample shape index; 2—sample height; 3—lateral lesions of the sample.

It can be seen from the diagram shown in Figure 4, that there is no linear progression (red line) in the process from the start of sintering of the calcium aluminoferrite sample to its complete melting.

The procedure for determination of the heat treatment temperature of variants containing a limited amount of liquid phase was as described below. As a first step, it was assumed that the process from the start of sintering to the complete melting of the sample could be represented as a line segment of length corresponding to 100% (*y*-axis). In the next step, it was determined that the heat treatment temperature corresponds to half the length of the line segment (50%) and then, by considering the profile of the curves, its position can be determined and then the desired temperature can be determined.

**Figure 5:**
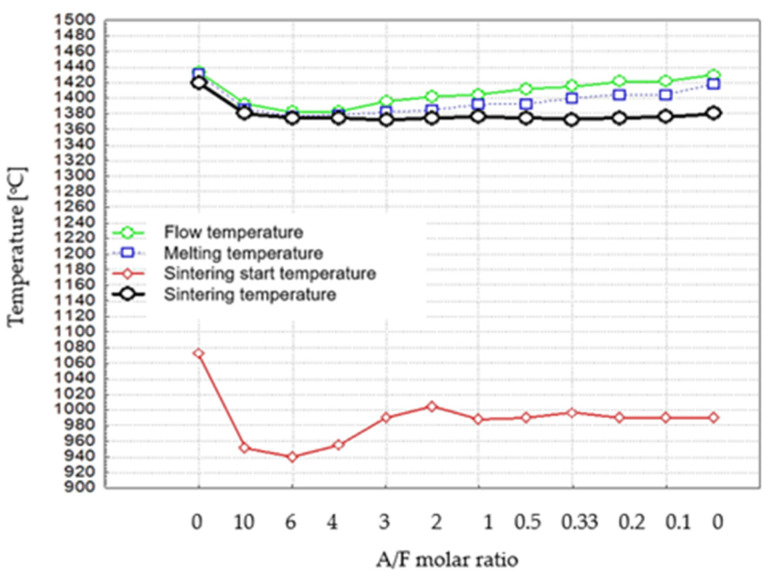
Dependence of characteristic temperatures of thermal processes on the chemical composition of the formulations.

The sintering, melting, and flow temperatures of the tested samples, determined as previously described, are summarised in Table 4 and Figure 5.

The results of the determination of the melting temperatures of the samples obtained in this study, after considering measurement errors, are in rough agreement with the literature data reported by Newkirk and Thwait [8], who determined melting temperatures for several phases (C_2_F—1438 °C, C_4_AF—1403 °C, and C_2_A about 1440 °C).

The data in Table 4 and Figure 5 indicate that there is a temperature interval of about 10 °C between the melting point and the flow temperature of samples covering the composition range from C_2_F to C_8_AF_3_. For samples with compositions ranging from C_2_A to C_10_A_4_, this interval is reduced. In addition, a temperature interval, around 15 °C, is observed between the temperature of the thermal treatment with a limited liquid phase and the melting temperature for the samples in the composition range from C_2_A to C_10_A_4_F. In contrast, for several samples from C_8_AF_3_ to C_2_F, this interval gradually increases to 50 °C. It can be seen that the gradual increase in the above interval is associated with an increase in Fe_2_O_3_ content in a series of successive thermally treated phases.

### 2.4. Method for XRD Phase Composition Studies

X-ray measurements were performed on samples with grain sizes below 0.020 mm. A vibratory agate mill (Frisch, Germany) was used to grind 3 g of samples and an additional 15% (by weight) of metallic Si (‘Silicon Powder’ SRM 640, X-ray Diffraction Standard, NIST, Gaithersburg, MD, USA). A grinding time of 1.5 h was applied. A vibrating mill with an external diameter of 95 mm and a grinding ball diameter of 50 mm was used; the weight of the grinding ball was 171 g. The vibration mill was characterised by an amplitude of 0.2 mm at a vibration frequency of 2000 Hz.

All samples without and with added standards were prepared in an analogous manner. Ground samples of approximately 1 g were placed in a flat measuring holder and immediately subjected to measurements to avoid prolonged contact with humidity.

XRD tests were carried out on a “PHILIPS” apparatus (Amsterdam, The Netherlands), consisting of a PW 1140/00/60 X-ray tube power supply and an upgraded PW 1050/50 vertical goniometer. The goniometer was equipped with software for fully automatic control with simultaneous digital recording of measurement data. The equipment setup included a vertically mounted ‘PHILIPS’ X-ray tube with cobalt anticathode (Co) and a wavelength of K_α_ = 1.7910 Å. An ‘Fe’ filter preventing the fluorescence effect from appearing in preparations containing large amounts of iron was used.

A PW 2216/20 ‘fine focus’ X-ray tube with a power of 1200 W and a window size of 0.4 × 8.0 mm and a focusing area of 3.2 mm^2^ was used, which, at an incidence angle of 6°, allowed the generation of a radiation beam with a width of 0.05 mm. The 1000 W lamp was operated at 40 kV with a cathode current of 25 mA. Measurements were carried out for a fixed angular range, from 10° to 80°2θ. For quantitative studies, a 20% addition of powdered metallic silicon was used with analogous powder preparation conditions.

Based on the X-ray data, a qualitative analysis of the samples was carried out to detect the presence of the relevant phases and a quantitative analysis to determine the mass proportion of crystalline and ‘amorphous’ phases, as well as the lattice parameters of the elementary cells of the detected phases. The Rietveld method [16,17,18] was used for quantitative data analysis. Specialised software such as X’PertHighScore Plus v. 2.1 from ‘Philips’ [19], and EXPGUI v. 3.0 from Los Alamos National Laboratory were used to process the data. In addition, the software “Jana 2006” from the Institute of Physics ASCR in Prague [20] and ANALYSIS-RayfleX v. 2.8 [21], as well as “MERCURY 3.7” Crystal Structure Visualisation (CCDC) [22,23] were also used.

## 3. Results

**Figure 6:**
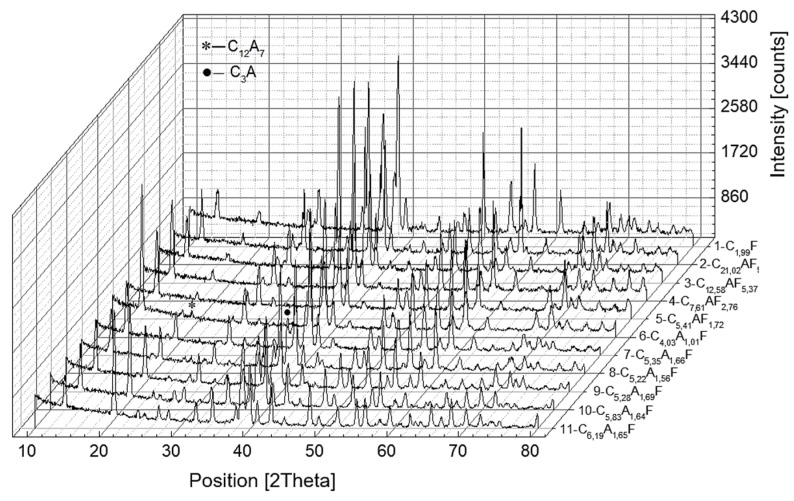
X-ray diffraction patterns registered for calcium aluminoferrites cooled at a slow rate.

**Figure 7:**
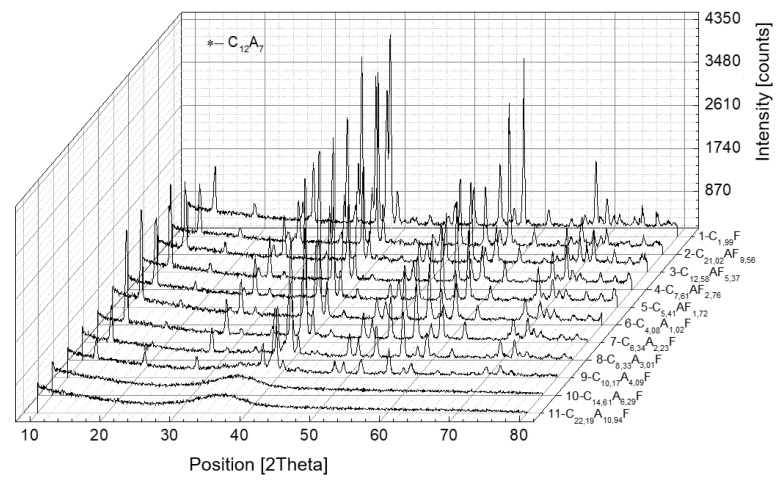
X-ray diffraction patterns of calcium aluminoferrites cooled at a rapid rate.

Figure 6 and Figure 7 summarize the X-ray diffractograms of the samples, which were subjected to slow or rapid cooling, respectively.

The results of the quantification of the phases formed are given in Table 5 and Table 6.

In samples numbered seven to eleven, the composition of the aluminoferrite phase is altered compared to the nominal composition and, in addition, for the C_x_A_y_F_z_ phase, the A/F ratio is approximately 1.64.

As the A/F molar ratio increases above one, an increase in the amount of mayenite is observed, with a simultaneous decrease in the concentration of calcium aluminoferrites. The C_3_A phase is separated only when the A/F molar ratio is above three. A further increase in the A/F ratio induces a decrease in the mass proportion of the C_3_A phase. For the samples subjected to rapid cooling, only the calcium aluminoferrite phase was found, with the exception of sample number nine with a composition of C_10.17_A_4.09_F, in which an amorphous phase was identified in addition to the crystalline calcium aluminoferrite phase and C_12_A_7_. In the last two samples, where the A/F molar ratio is six and ten, only amorphous calcium aluminoferrite is present.

Table 7 and Table 8 summarise the determined interplane distances *d* and the corresponding intensities as a function of the A/F molar ratio.

From these data, it can be seen that as the Al_2_O_3_ content of the calcium aluminoferrite phases increases, there is a decrease in the *d* values. For example, for the plane (141), to which the highest intensity corresponds, the *d* values vary from 2.680 Å to 2.618 Å.

**Figure 8:**
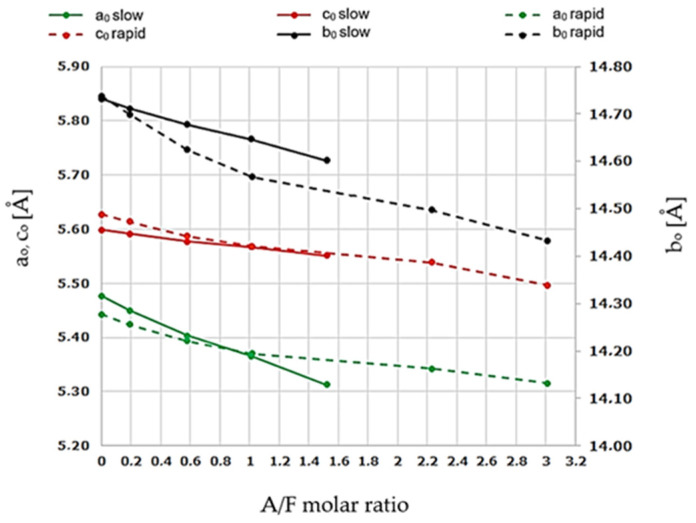
Changes in elemental cell dimensions of calcium aluminoferrites as a function of the A/F molar ratio after slow and rapid cooling.

**Figure 9:**
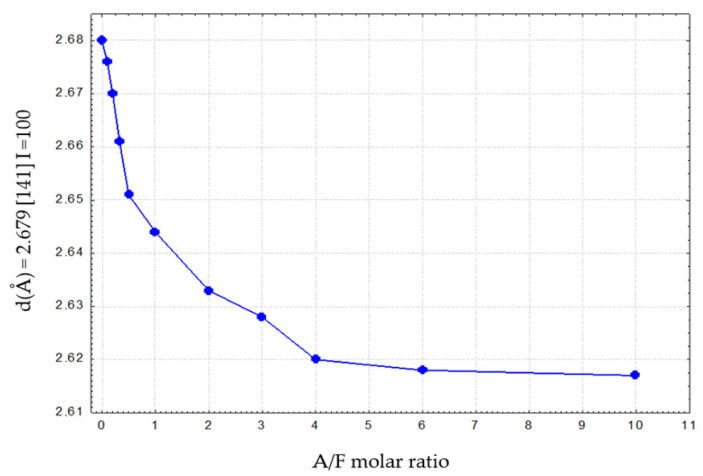
Changes in *d* values for reflex (141) vs. A/F ratio for calcium aluminoferrites cooled at a slow rate.

Figure 8 and Figure 9 illustrate the variation of *d* for reflex (141) as a function of the Al_2_O_3_/Fe_2_O_3_ molar ratio for calcium aluminoferrite subjected to slow or rapid cooling.

These relations show an approximately linear course and can, therefore, be described by Vegard’s rule [23,24]. The fit of the experimental data for slowly cooled samples is better compared to rapidly cooled samples. This can be explained by the slow formation of the crystal structure of these samples. For samples subjected to rapid cooling, the weaker fit of the linear relationship is due to the need for immediate crystallisation of the phases from the melt and the proportion of the amorphous phase in the test samples. Deviations from the Vegard rule relate, among other things, to the case of systems that do not exhibit isomorphism. The appearance of additional crystalline phases in the calcium aluminoferrite solid solutions studied results in a deviation from the Vegard rule [24,25]. The noticeable changes in the interplanar distances ‘d’ of the calcium aluminoferrites phase are due to changes in their elemental cell parameters, most likely as a result of differences in the ionic radiuses of the aluminium and iron (III) cations [24,25].

The results of the determinations of the elemental cell parameters of the calcium aluminoferrite phases subjected to slow and rapid cooling are included in Table 9.

**Figure 10:**
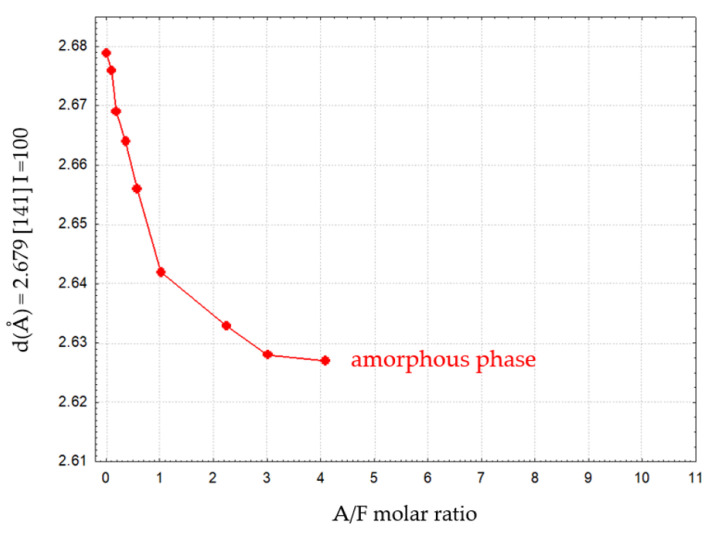
Changes in *d* values for reflex (141) vs. A/F ratio for calcium aluminoferrites cooled at a rapid rate.

These values are in good agreement with the literature data [8,9] and follow the interpretation related to the phenomenon of shifting extremes of the calcium aluminoferrite phase peaks towards higher 2θ angles. Analysis of the data in Table 9 indicates changes in the linear dimensions of the elemental cells along all a_0_, b_0_, and c_0_ axes, which translates into changes in the volume of the V_roent_. cells and their X-ray densities ρ_roent_. Figure 9 and Figure 10 illustrate the range of differences in the dimensions of the a_0_, b_0_, and c_0_ axes of the elemental cells of the examined phases as a function of changes in the A/F ratio and the conditions of the cooling process of the studied samples.

A decrease in all elementary cell dimensions is observed. The largest changes were recorded for the a_0_ and c_0_ axes and the smallest for the b_0_ axis.

The results show that changes in the linear dimensions of the a_0_, b_0_, and c_0_ axes of the aluminoferrites elemental cells occur with an increase in the A/F ratio in the range of values from 0.10 to 4.09 and include a simultaneous decrease in the linear dimensions on all three crystallographic axes. As can be seen in Figure 9 and Figure 10, the nature of the above changes is nearly linear, and the underlying reasons for the deviation should be sought in measurement errors. It can also be seen that the above dependencies are similar for the a_0_ and b_0_ axes, while they differ for the c_0_ axis. The range of changes in the linear dimensions of the elemental cells of calcium aluminoferrites, for which the A/F ratio varies from 0.1 to 4.09, is for axes a_0_ = 0.228 Å, c_0_ = 0.092 Å, and b_0_ = 0.204 Å. As shown in numerous studies [8,9,10,11,12], the reason for these changes is the substitution of Fe^3+^ ions by Al^3+^ ions, which have a smaller ionic diameter than that of iron (III) ions. After rapid crystallisation of the sample from the melt, the phenomenon of a shift in the extremes of all the peaks originating from the calcium aluminoferrites phases towards higher 2θ angles can be observed.

The variation of the ‘d’ parameters for the (141) peak with *I* = 100% as a function of the A/F molar ratio in calcium aluminoferrites previously melted and then rapidly cooled is shown in Table 8. Again, with the appearance of amorphous phases, the nature of the ‘truncated’ curve illustrating this relationship indicates the presence of calcium aluminoferrite phases that belong to solid solutions. Above this A/F ratio of >4.01, amorphous phases are released.

The correlations presented above indicate that the changes in the parameters of the a_0_, b_0_, and c_0_ axes of elemental aluminoferrite cells subjected to melting and then rapid cooling follow the same principle of a decrease in the linear dimensions of all three axes with an increase in the A/F ratio of the studied phases. As can be seen from Figure 9, the changes for the a_0_ and c_0_ axes follow an almost linear course and the course of changes in linear dimensions for the longest b_0_ axis is different. The range of changes of the cells of calcium aluminoferrites, for which the A/F ratio varies from 0.1 to 4.09, is, for the a_0_ and c_0_ axes, approximately 0.148 Å and 0.155 Å, respectively, and for the b_0_ axis = 0.346 Å.

The results obtained show that the dimensions of the aluminoferrite elementary cells undergo significant changes when the melts are rapidly cooled. If we compare these results with the decrease in the linear dimensions of the elemental cell axes of aluminoferrites slowly cooled as a result of the substitution of Al^3+^ for Fe^3+^, the differences recorded must be considered to be very large. These differences are for the axes a_0_ = 0.1818 Å, c_0_ = 0.1273 Å, and b_0_ = 0.3404 Å and are large enough to be the result of additional deformation and distortion of the elemental cells of the calcium aluminoferrite phases subjected to rapid cooling.

The results of the study allow us to conclude that in all the analysed samples, irrespective of the cooling method, the presence of unreacted oxides of CaO, α-Al_2_O_3_, Fe_2_O_3_, and metallic iron was not identified in the phase composition of the samples. The absence of these oxides in the mineral compositions of the melts should be related to the fact that the heat-treated samples fully reacted under the appropriate process conditions.

As a result of the melting of the samples, irrespective of the applied cooling method, only calcium aluminoferrites with varying chemical compositions formed for an A/F molar ratio from 0.10 to 1.02. The formation of these phases in the given A/F ratio range is not accompanied by the simultaneous release of other additional compounds. Based on an in-depth analysis of the changes in the elementary cell parameters of the phases, they were considered to belong to the C_6_A_x_F_3−x_ group where 0 < x < 3 of the calcium aluminoferrites of the solid solution family occurring between C_2_F and the hypothetical C_2_A.

Slow cooling of calcium aluminoferrite melts characterised by an A/F ratio ≥ 2.23 led to the separation of two or three different phases. As the A/F ratio increased from 1.02 to 10.94, a calcium aluminoferrite phase with a slightly varying chemical composition was formed in the analysed samples in addition to mayenite (C_12_A_7_) and tricalcium aluminate (C_3_A).

Calculations show that its composition, treated as an ‘average composition’, is close to that of the phase with formula C_5,8_A_1,7_F. It appears that, in the analysed area of variation of the A/F ratio, the content of the mayenite phase increases to a value of about 65% (wt.) and, in the extreme case, the C_3_A level reaches about 12% (wt.).

Qualitative and quantitative analysis of the phase compositions of the samples subjected to rapid cooling with A/F molar ratios ranging between 0.10 and 4.09 indicates the presence of only an aluminoferrite phase of variable chemical composition, which is free of admixtures of other compounds. The previously mentioned area of A/F ratio variation covers about one-third of the total study range. In contrast to all other rapidly cooled preparations, for which the A/F ratio is equal to 4.09, mayenite and a glassy phase are released in addition to calcium aluminoferrites. Rapid cooling of the melts with a further increase in the A/F molar ratio above 4.09 contributes to a change in the physical state and a transition of the preparations to an amorphous form.

It can be concluded that by using the correct synthesis and cooling procedure, the phase composition of the resulting melts can be influenced in a controlled manner. In the case of the present work, synthesis by melting and rapid cooling of the melts led to an aluminoferrite phase that was free of admixtures of other compounds in the range of A/F molar ratios from 1.02 to 4.09.

X-ray studies confirmed the low efficiency of obtaining good quality calcium aluminoferrite preparations during a long-time synthesis, lasting 6 h, based on sintering at a temperature approximately 40 °C lower than the melting point of the samples.

Based on the observation of the phenomenon of shifting peak extremes originating from calcium aluminoferrites and the analysis of changes in elementary cell parameters due to changes in the A/F ratio, it can be concluded that the studied phases belong to the family of solid solutions of calcium aluminoferrites of the C_6_A_x_F_3−x_ group, where 0 < x < 3.

The study linked a decrease in the linear dimensions of the elemental cells of calcium aluminoferrite solid solutions to an increase in their Al_2_O_3_ content. Considering the above, the formation of compounds belonging to the family of calcium aluminoferrite solid solutions described by the formula C_6_A_x_F_3−x_, where 0 < x < 3 should be associated with the effect of substitution in the C_2_F elementary cell of some Fe^3+^ ions with Al^3+^ ions of smaller ionic radius [2,8].

It also seems reasonable that with changes in the linear dimensions of the a_0_, b_0_, and c_0_ axes, while maintaining the same crystallographic arrangement, there will be a change in the volume of the elemental cell and its X-ray density.

The changes in the relationship between the linear dimensions of the calcium aluminoferrite elemental cells and the A/F molar ratio of the melted preparations, which were subjected to slow and rapid cooling, as discussed above, indicate the existence of differences that increase with increasing A/F molar ratio in the aluminoferrite phases.

## 4. Discussion

This is the first known study concerning the changes in the phase composition of calcium aluminoferrites depending on the synthesis conditions and the selection of the Al_2_O_3_/Fe_2_O_3_ molar ratio (A/F), which extends beyond the limiting composition of C_6_A_2_F towards phases richer in Al_2_O_3_. There is practically no, or scarce, literature data on this subject.

As a result of the application of an appropriate thermal treatment procedure and cooling of the preparations, a series of phases belonging to the calcium aluminoferrite group were obtained, described by the formula C_6_A_x_F_3−x_, where 0 < x < 3, in which the molar ratio A/F varied from 0.10 to 10.94, while the constant molar ratio CaO/Al_2_O_3_ + Fe_2_O_3_ of 2.0 was kept.

The qualitative and quantitative roentgenographic studies that were carried out confirmed the different phase compositions of the preparations obtained, in that in some cases samples were obtained consisting of only calcium aluminoferrites, or multiphase preparations consisting of a mixture of mayenite (C_12_A_7_), tricalcium aluminate (C_3_A), and (C_x_A_y_F_z_) calcium aluminoferrite phase. In extreme cases, partially or completely amorphous preparations were obtained. It was proven that the phase composition of the samples depended primarily on the A/F molar ratio, which characterises the oxide contributions in the sample, as well as on the applied thermal treatment.

Slowly cooled samples were characterised by the appearance of phases derived exclusively from calcium aluminoferrites when the molar ratio A/F was in the range of 0.10 to 0.58. A further increase in this A/F ratio range from 0.58 to 2.23 favoured the appearance of mayenite (C_12_A_7_) in addition to the calcium aluminoferrite phase. In the range of changes in the A/F molar ratio from 3.01 to 10.94, the co-occurrence of C_12_A_7_, C_3_A, and the aluminoferrite phase is observed. From the X-ray quantitative analysis, it can be concluded that, with an increase in the A/F ratio from 1.02 to 10.94, there is a successive decrease in the mass proportion of the aluminoferrite phase (from 99.5% to 26.4%) with a concomitant increase in the amount of mayenite (from 0.5% to 65.6%). Furthermore, these changes are accompanied by a decrease in C_3_A content from 13.2% to 8.0%.

The cell parameters of the elementary phases of calcium aluminoferrites, determined using the Rietveld method, showed that there is a gradual decrease in the linear dimensions of the cells in the region of single-phase aluminoferrite existence (A/F changes from 0.10 to 0.58) and the difference in these changes with respect to the individual axes is a_0_ = 0.228 Å, b_0_ = 0.204 Å, and c_0_ = 0.092 Å. The observed changes in the linear dimensions of the elemental cells are a result of the substitution of Fe^3+^ ions with Al^3+^ ions of smaller ionic radius in calcium aluminoferrites characterised by variations in the A/F ratio.

The phase compositions of rapidly cooled samples behave differently compared to the slowly cooled samples. In the range of an A/F molar ratio from 0.10 to 3.01 (inclusive), the analysed preparations consist of 100% aluminoferrite phases. It should be noted that, in this case, the range of single-phase calcium aluminoferrite phases was extended from A/F = 2.23 to a level of A/F = 3.01. At an A/F ratio of 4.09% mayenite, 50% aluminoferrite, and 40% of an amorphous phase co-occur. With a further increase in the A/F molar ratio between 4.09 and 10.94, there is a change in the physical state of the samples due to their transformation to the amorphous form.

Relationships between changes in the linear dimensions of the elemental cell axes of calcium aluminoferrites resulting from changes in the A/F molar ratio during melting and slow or rapid cooling indicate significant differences, which increase with increasing A/F ratios in the studied phases.

Upon rapid cooling of calcium aluminoferrite samples, the linear dimensions of the elementary cell phase axes shorten for the a_0_ axis by about 0.071 Å, with a concomitant lengthening of the c_0_ axis by about 0.060 Å and of the b_0_ axis by about 0.143 Å. The above leads to the conclusion that during rapid cooling of calcium aluminoferrite-based melts, structural deformations occur that favour an increase in the degree of defectivity of the crystalline structure of the studied phases, which may lead to an increase in their chemical reactivity in various reactions environments. When the A/F molar ratio is kept between 4.09 and 10.94, partially or completely amorphous phases are released in the preparations obtained after rapid cooling.

This study comprises the character of basic research, as it focuses on the influence of the admixing of calcium ferrate phases with aluminium ions. The analysis of the calcium aluminoferrites line as well as the extent to which their solid solutions occur on the C_2_F–“C_2_A” line, due to the occurrence of this type of phase in aluminous cements as well as in Portland cements from the point of view of cement chemistry and its technology, is a key issue. An appropriate modelling of the calcium aluminoferrite phases can contribute to the preparation of a stand-alone binder or activator for the setting and hardening of Portland cements.

Concerning future practical implications, the method of synthesis and appropriately selected waste materials (as additives) will lead to the production of an alternative binder with the characteristics of aluminous cements, such as fondu. In further stages, it is planned to analyse the hydration activity of the calcium aluminoferrites obtained in processes involving water.

Produced samples will be subjected to hydraulic activity tests. Tests will be performed towards calorimetric analysis of the hydrating slurries and the influence of cooling conditions and the chemical composition of the calcium aluminoferrite phases and their mixtures. Selected samples of the most active phases will be tested in mixtures with Portland cement with low C_3_A contents. Pastes from different phases of calcium aluminoferrites and their mixtures, as well as the mixtures of calcium aluminoferrites with Portland cement, will be analysed for mechanical strength and durability against chemical corrosion.

## 5. Conclusions

The results of this study allowed for the following conclusions:

1. A significant effect of the conditions under which calcium aluminoferrites were obtained on their phase composition was observed.
2. In slowly cooled samples with A/F ratios of 0.10 to 0.58, calcium aluminoferrite phases are released, while in other cases calcium aluminoferrites co-occur with C_12_A_7_ and C_3_A.
3. Under rapid cooling conditions for samples with A/F ratios between 0.10 and 3.01, only calcium aluminoferrites are formed. In the case of the sample with a composition of C_10.17_A_4.09_F, calcium aluminoferrite co-occurs with C_12_A_7_ calcium aluminate and an amorphous phase. Samples with A/F ratios between 6.29 and 10.94 are amorphous.
4. Quantitative roentgenographic studies of calcium aluminoferrites have shown that changes in elemental cell parameters are a consequence of the cooling conditions used and changes in the A/F molar ratio. Significant structural changes are observed for samples subjected to rapid cooling.

## Figures and Tables

**Table 1:** Elementary cell parameters of a sequence of solid solutions of the C_x_A_y_F_z_ type [2].

Phase	Parameters of the Elementary Cell [Å,^o^]				Elementary Cell Volume [Å^3^] ±0.1
	a	b	c	α; β; γ = 90	
C_6_A_2.07_F_0.93_	5.513	14.484	5.309	90	423.9
C_6_A_2_F	5.480	14.350	5.220	-	-
	5.526	14.469	5.314	90	424.8
C_6_A_1.75_F_1.25_	5.548	14.490	5.331	90	428.5
C_6_A_1.5_F_1.5_	5.510	14.420	5.260	-	-
	5.580	14.500	5.340	90	432.1
	5.584	14.600	5.374	-	438.1
	5.567	14.521	5.349	90	432.4
	5.565	14.514	5.346	90	431.8
C_6_A_1.25_F_1.75_	5.579	14.550 ± 0.002	5.361	90	435.1
C_6_A_1.09_F_1.91_	5.583	14.580	5.374	90	437.4
C_6_AF_2_	5.580	14.600	5.370	90	437.5
	5.588	14.587 ± 0.002	5.375	90	438.1
C_6_A_0.9_F_2.1_	5.588	14.610	5.38	90	439.2
C_6_A_0.5_F_2.5_	5.595	14.679	5.403	90	443.7
C_6_F_3_ (C_2_F)	5.580	14.630	5.320	-	446.7
	5.640	14.680	5.390	-	-
	5.599	14.771	5.429	-	449.0
	5.600	14.767	5.428	90	448.8

**Table 2:** Nominal composition of calcium aluminoferrites.

Item	Nominal Composition	Oxide Composition [% Weight]			Mass Ratio Al_2_O_3_/Fe_2_O_3_	Molar Ratio Al_2_O_3_/Fe_2_O_3_	Molar Ratio CaO/(Al_2_O_3_ + Fe_2_O_3_)
		CaO	Al_2_O_3_	Fe_2_O_3_			
1.	C_2_F	41.25	-	58.75	-	-	2.0
2.	C_22_AF_10_	42.07	3.48	54.45	0.06	0.10	2.0
3.	C_12_AF_5_	42.77	6.48	50.75	0.13	0.20	2.0
4.	C_8_AF_3_	43.57	9.90	46.53	0.21	0.33	2.0
5.	C_6_AF_2_	44.40	13.45	42.15	0.32	0.50	2.0
6.	C_4_AF	46.16	20.98	32.86	0,64	1.00	2.0
7.	C_6_A_2_F	48.06	29.13	22.81	1.28	2.00	2.0
8.	C_8_A_3_F	49.07	33.46	17.47	1.92	3.00	2.0
9.	C_10_A_4_F	49.70	36.15	14.15	2.55	4.00	2.0
10.	C_14_A_6_F	50.44	39.30	10.26	3.83	6.00	2.0
11.	C_22_A_10_F	51.13	42.25	6.62	6.38	10.00	2.0

**Table 3:** Chemical composition of the synthesized calcium aluminoferrites.

Item	Nominal Composition	Oxide Composition [% Weight]			Actual Composition
		CaO	Al_2_O_3_	Fe_2_O_3_	
1.	C_2_F	40.95	-	58.55	C_1.99_F
2.	C_22_AF_10_	41.97	3.63	54.40	C_21.02_AF_9.56_
3.	C_12_AF_5_	42.35	6.12	51.53	C_12.58_AF_5.37_
4.	C_8_AF_3_	43.95	10.51	45.54	C_7.61_AF_2.76_
5.	C_6_AF_2_	44.65	15.00	40.35	C_5.41_AF_1.72_
6.	C_4_AF	46.45	21.15	32.40	C_4.08_A_1.02_F
7.	C_6_A_2_F	47.90	30.60	21.50	C_6.34_A_2.23_F
8.	C_8_A_3_F	50.00	32.90	17.10	C_8.33_A_3.01_F
9.	C_10_A_4_F	49.69	36.40	13.91	C_10.17_A_4.09_F
10.	C_14_A_6_F	50.55	39.60	9.85	C_14.61_A_6.29_F
11.	C_22_A_10_F	50.50	43.30	6.20	C_22.19_A_10.94_F

**Table 4:** The sintering, melting, and flow temperatures of the examined calcium aluminoferrites.

Phases with a Composition		Characteristic Temperatures [°C]			
Nominal	Actual	Start of Sintering (Experimental)	Start of Sintering (Calculated)	Melting	Flow
C_2_F	C_1.99_F	990	1380	1418	1430
C_22_AF_10_	C_21.02_AF_9.56_	990	1376	1405	1422
C_12_AF_5_	C_12.58_AF_5.37_	990	1375	1405	1421
C_8_AF_3_	C_7.61_AF_2.76_	997	1373	1400	1415
C_6_AF_2_	C_5.41_AF_1.72_	990	1375	1383	1412
C_4_AF	C_4.08_A_1.02_F	988	1376	1392	1405
C_6_A_2_F	C_6.34_A_2.23_F	1005	1375	1385	1402
C_8_A_3_F	C_8.33_A_3.01_F	990	1372	1382	1396
C_10_A_4_F	C_10.17_A_4.09_F	955	1375	1379	1383
C_14_A_6_F	C_14.61_A_6.29_F	940	1375	1377	1382
C_22_A_10_F	C_22.19_A_10.94_F	951	1380	1386	1393
“C_2_A”	C_1.96_A_1.04_	1072	1420	1431	1434

Note: The error of the method used is [±2.5 °C].

**Table 5:** Composition of C_x_A_y_F_z_ for calcium aluminoferrites cooled at a slow rate.

Item	Actual Composition	Molar Ratio Al_2_O_3_/Fe_2_O_3_	Phase Composition [% Weight]			Molar Phase Composition C_x_A_y_F_z_			Y/Z
			C_12_A_7_	C_3_A	C_x_A_y_F_z_	X	Y	Z	
1.	C_1.99_F	-	-	-	-	1.991	-	-	-
2.	C_21.02_AF_9.56_	0.10	-	-	100.0	21.021	1.000	9.568	0.10
3.	C_12.58_AF_5.37_	0.19	-	-	100.0	12.582	1.000	5.375	0.19
4.	C_7,61_AF_2.766_	0.36	-	-	100.0	7.603	1.000	2.766	0.36
5.	C_5.41_AF_1.72_	0.58	-	-	100.0	5.412	1.000	1.717	0.58
6.	C_4.08_A_1.02_F	1.02	0.5	-	99.5	4.030	1.009	1.0	1.00
7.	C_6.34_A_2.23_F	2.23	15.0	-	85.0	5.353	1.666	1.0	1.67
8.	C_8.33_A_3.01_F	3.01	21.1	13.2	65.7	5.218	1.562	1.0	1.56
9.	C_10.17_A_4.09_F	4.09	32.8	12.0	55.2	5.283	1.687	1.0	1.69
10.	C_14.61_A_6.29_F	6.29	50.5	8.8	40.7	5.828	1.636	1.0	1.64
11.	C_22.19_A_10.94_F	10.94	65.6	8.0	26.4	6.191	1.646	1.0	1.65

**Table 6:** Composition of C_x_A_y_F_z_ for calcium aluminoferrites cooled at a rapid rate.

Item	Actual Composition	Molar Ratio Al_2_O_3_/Fe_2_O_3_	Phase Composition [% Weight]				Molar Phase Composition C_x_A_y_F_z_			Y/Z
			C_12_A_7_	C_3_A	C_x_A_y_F_z_	Amorphous	X	Y	Z	
1.	C_1.99_F	-	-	-	-	-	1.991	-	-	-
2.	C_21.02_AF_9.56_	0.10	-	-	100.0	-	21.021	1.000	9.568	0.10
3.	C_12.58_AF_5.37_	0.19	-	-	100.0	-	12.582	1.000	5.375	0.19
4.	C_7.61_AF_2.766_	0.36	-	-	100.0	-	7.603	1.000	2.766	0.36
5.	C_5.41_AF_1.72_	0.58	-	-	100.0	-	5.412	1.000	1.717	0.58
6.	C_4.08_A_1.02_F	1.02	-	-	100.0	-	4.030	1.009	1.000	1.00
7.	C_6.34_A_2.23_F	2.23	-	-	100.0	-	6.337	2.226	1.000	2.23
8.	C_8.33_A_3.01_F	3.01	-	-	100.0	-	8.329	3.010	1.000	3.01
9.	C_10.17_A_4.09_F	4.09	8.9	-	50.1	41	-	-	-	4.09
10.	C_14.61_A_6.29_F	6.29	-	-	-	100	-	-	-	6.29
11.	C_22.19_A_10.94_F	10.94	-	-	-	100	-	-	-	10.90

**Table 7:** Values of *d* and intensity *I* * for calcium aluminoferrites cooled at a slow rate.

**Phase**	***h k l***	**1**		**2**		**3**		**4**		**5**		**6**	
		**C_1.99_F**		**C_21.02_AF_9.56_**		**C_12.58_AF_5.37_**		**C_7.61_AF_2.76_**		**C_5.41_AF_1.72_**		**C_4.03_A_1.01_F**	
		***d*** **[Ǻ]**	***I*** **[%]**	***d*** **[Ǻ]**	***I*** **[%]**	***d*** **[Ǻ]**	***I*** **[%]**	***d*** **[Ǻ]**	***I*** **[%]**	***d*** **[Ǻ]**	***I*** **[%]**	***d*** **[Ǻ]**	***I*** **[%]**
ferrite	*0 2 0*	7.431	15	7.409	28	7.377	21	7.338	38	7.299	48	7.265	64
C_12_A_7_	*2 1 1*	-	-	-	-	-	-	-	-	-	-	4.899	10
ferrite	-	-	-	-	-	-	-	-	-	-	-	-	-
ferrite	*1 3 0*	3.708	18	3.694	33	3.683	22	3.673	22	3.659	28	3.646	20
ferrite	*-*	-	-	-	-	-	-	-	-	-	-	-	-
ferrite	*-*	-	-	-	-	-	-	-	-	-	-	-	-
ferrite	*2 0 0*	2.808	37	2.805	47	2.800	52	2.798	28	2.792	47	2.783	36
C_3_A	*4 4 0*	-	-	-	-	-	-	-	-	-	-	-	-
ferrite	*0 0 2*	2.709	80	2.701	50	2.694	59	2.691	32	2.685	50	2.677	39
ferrite	*1 4 1*	2.680	100	2.676	100	2.670	100	2.661	100	2.651	100	2.644	100
ferrite	*1 5 0*	2.613	14	2.606	20	2.601	26	2.592	20	2.578	38	2.573	20
ferrite	*2 1 1*	2.451	15	2.449	15	2.446	10	2.444	10	2.441	15	2.439	10
ferrite	*2 4 0*	2.234	10	2.228	10	2.224	12	2.217	15	2.214	10	2.209	12
ferrite	*0 4 2*	2.188	10	2.186	18	2.180	10	2.169	12	2.164	18	2.155	11
ferrite	*1 6 1*	2.082	16	2.076	26	2.071	27	2.062	28	2.054	36	2.048	37
ferrite	-	-	-	-	-	-	-	-	-	-	-	-	-
ferrite	*2 2 2*	1.908	10	1.901	13	1.897	10	1.889	10	1.873	13	1.865	10
ferrite	*3 3 0*	1.747	20	1.745	10	1.744	11	1.741	10	1.735	15	1.732	11
		**7**		**8**		**9**		**10**		**11**			
**Phase**	***h k l***	**C_5.35_A_1.66_F**		**C_5.22_A_1.56_F**		**C_5.28_A_1.68_F**		**C_5.83_A_1.64_F**		**C_6.19_A_1.65_F**			
		***d*** **[Ǻ]**	***I*** **[%]**	***d*** **[Ǻ]**	***I*** **[%]**	***d*** **[Ǻ]**	***I*** **[%]**	***d*** **[Ǻ]**	***I*** **[%]**	***d*** **[Ǻ]**	***I*** **[%]**		
ferrite	*0 2 0*	7.248	38	7.224	62	7.199	47	7.192	57	7.194	32		
C_12_A_7_	*2 1 1*	4.903	17	4.906	35	4.884	45	4.878	100	4.894	100		
ferrite	-	-	-	-	-	-	-	3.779	15	3.794	13		
ferrite	*1 3 0*	3.639	22	3.632	20	3.620	22	3.613	21	3.615	12		
ferrite	-	-	-	-	-	-	-	3.199	20	3.202	20		
ferrite	-	-	-	-	-	-	-	2.994	31	2.995	39		
ferrite	*2 0 0*	2.770	34	2.762	38	2.759	58	2.757	32	2.758	19		
C_3_A	*4 4 0*	-	-	2.698	27	2.683	30	2.674	32	2.762	27		
ferrite	*0 0 2*	2.659	36	2.653	42	2.651	40	2.649	79	2.652	76		
ferrite	*1 4 1*	2.633	78	2.628	100	2.621	84	2.618	84	2.616	49		
ferrite	*1 5 0*	2.565	30	2.561	22	2.554	22	2.553	27	2.556	23		
ferrite	*2 1 1*	2.435	10	2.429	15	2.420	20	2.419	37	2.421	38		
ferrite	*2 4 0*	2.198	15	2.194	17	2.186	30	2.186	45	2.189	35		
ferrite	*0 4 2*	2.146	12	2.142	14	2.137	18	2.135	20	2.138	11		
ferrite	*1 6 1*	2.042	21	2.039	60	2.033	31	2.031	50	2.033	19		
ferrite	*-*	-	-	-	-	-	-	1.904	42	1.909	35		
ferrite	*2 2 2*	1.853	10	1.851	13	1.847	13	1.849	14	-	-		
ferrite	*3 3 0*	1.726	10	1.721	13	1.722	13	1.724	11	-	-		

* Note: In accordance with the ICDD Commission guidelines, only reflections with an intensity of more than 10% are included.

**Table 8:** Values of *d* and intensity *I* * for calcium aluminoferrites cooled at a rapid rate.

**Phase**	***h k l***	**1**		**2**		**3**		**4**		**5**		**6**	
		**C_1.99_F**		**C_21.02_AF_9.56_**		**C_12.58_AF_5.37_**		**C_7.61_AF_2.76_**		**C_5.41_AF_1.72_**		**C_4.08_A_1.02_F**	
		***d*** **[Ǻ]**	***I*** **[%]**	***d*** **[Ǻ]**	***I*** **[%]**	***d*** **[Ǻ]**	***I*** **[%]**	***d*** **[Ǻ]**	***I*** **[%]**	***d*** **[Ǻ]**	***I*** **[%]**	***d*** **[Ǻ]**	***I*** **[%]**
ferrite	*0 2 0*	7.431	15	7.409	30	7.369	64	7.349	47	7.289	52	7.255	50
C_12_A_7_	*2 1 1*	-	-	-	-	-	-	-	-	-	-	-	-
ferrite	-	-	-	-	-	-	-	-	-	-	-	-	-
ferrite	*1 3 0*	3.695	47	3.693	26	3.686	21	3.678	22	3.662	24	3.652	17
ferrite	-	-	-	-	-	-	-	-	-	-	-	-	-
ferrite	-	-	-	-	-	-	-	-	-	-	-	-	-
ferrite	*2 0 0*	2.804	48	2.803	51	2.805	29	2.801	44	2.793	33	2.778	36
ferrite	*0 0 2*	2.716	60	2.715	34	2.703	44	2.694	47	2.681	30	2.674	37
ferrite	*1 4 1*	2.679	100	2.676	100	2.669	100	2.664	100	2.656	100	2.642	100
ferrite	*1 5 0*	2.614	13	2.611	28	2.601	14	2.595	20	2.584	12	2.574	14
ferrite	*2 1 1*	2.450	10	2.448	15	2.445	10	2.443	15	2.439	10	2.436	10
ferrite	*2 4 0*	2.229	10	2.214	10	2.209	12	2.194	17	2.219	10	2.208	11
ferrite	*0 4 2*	2.187	13	2.184	13	2.155	11	2.136	15	2.167	12	2.163	11
ferrite	*1 6 1*	2.080	21	2.075	40	2.071	21	2.065	24	2.059	23	2.048	22
ferrite	-	-	-	-	-	-	-	-	-	-	-	-	-
ferrite	*2 2 2*	1.906	24	1.899	83	1.895	18	1.890	21	1.874	10	1.862	10
ferrite	*3 3 0*	1.745	10	1.745	15	1.744	11	1.743	10	1.739	10	1.732	11
		**7**		**8**		**9**		**10**		**11**			
**Phase**	***h k l***	**C_6.34_A_2.23_F**		**C_8.33_A_3.01_F**		**C_10.17_A_4.09_F**		C_14.61_A_6.29_F **		C_22.19_A_10.94_F **			
		***d*** **[Ǻ]**	***I*** **[%]**	***d*** **[Ǻ]**	***I*** **[%]**	***d*** **[Ǻ]**	***I*** **[%]**	***d*** **[Ǻ]**	***I*** **[%]**	***d*** **[Ǻ]**	***I*** **[%]**		
ferrite	*0 2 0*	7.242	67	7.240	62	7.237	34	-	-	-	-		
C_12_A_7_	*2 1 1*	-	-	-	-	4.883	20	-	-	-	-		
ferrite	-	-	-	-	-	-	-	-	-	-	-		
ferrite	*1 3 0*	3.637	25	3.634	20	3.636	22	-	-	-	-		
ferrite	*-*	-	-	-	-	-	-	-	-	-	-		
ferrite	*-*	-	-	-	-	-	-	-	-	-	-		
ferrite	*2 0 0*	2.766	44	2.760	38	2.758	47	-	-	-	-		
ferrite	*0 0 2*	2.659	38	2.654	42	2.655	24	-	-	-	-		
ferrite	*1 4 1*	2.633	100	2.628	100	2.629	100	-	-	-	-		
ferrite	*1 5 0*	2.566	25	2.562	22	2.563	18	-	-	-	-		
ferrite	*2 1 1*	2.420	10	2.419	15	2.420	12	-	-	-	-		
ferrite	*2 4 0*	2.200	17	2.195	17	2.196	24	-	-	-	-		
ferrite	*0 4 2*	2.145	13	2.142	14	2.143	20	-	-	-	-		
ferrite	*1 6 1*	2.043	28	2.041	60	2.044	26	-	-	-	-		
ferrite	-	-	-	-	-	-	-	-	-	-	-		
ferrite	*2 2 2*	1.855	10	1.851	13	1.852	13	-	-	-	-		
ferrite	*3 3 0*	1.725	10	1.723	13	1.724	10	-	-	-	-		

* In accordance with the ICDD Commission guidelines, reflections with an intensity of more than 10% are included. ** Samples with compositions of C_22.19_A_10.94_F and C_14.61_A_6.29_F were characterised by the presence of an amorphous phase as a result of rapid cooling.

**Table 9:** Values of elemental cell lattice parameters for calcium aluminoferrites after slow and rapid cooling.

Sample No.	Phase	Cell Parameters in [Å] ±0.0040				V_roent._ [Å^3^]	ρ_roent._	Crystallographic System
		a_0_ [Å]	c_0_ [Å]	b_0_ [Å]	α = β = γ [^o^]			
Samples subjected to slow cooling								
1	C_1.99_F	5.4765	5.5989	14.7322	90	451.7242	3.9890	orthorhombic
3	C_12.58_AF_5.37_	5.4495	5.5911	14.7112	90	448.2311	3.9190	orthorhombic
5	C_5.41_AF_1.72_	5.4031	5.5775	14.6776	90	442.3210	3.7670	orthorhombic
6	C_4.08_A_1.02_F	5.3654	5.5673	14.6466	90	437.5055	3.6800	orthorhombic
7	C_6.34_A_2.23_F	5.3133	5.5515	14.6016	90	430.7002	3.5990	orthorhombic
8	C_8.33_A_3.01_F	5.2727	5.5223	14.5552	90	423.8100	3.5900	orthorhombic
9	C_10.17_A_4.09_F	5.2489	5.5070	14.5279	90	419.9390	3.5660	orthorhombic
Samples subjected to rapid cooling								
1	C_1.99_F	5.4424	5.6272	14.7382	90	451.3643	3.9920	orthorhombic
3	C_12.58_AF_5.37_	5.4238	5.6140	14.6997	90	447.5942	3.9240	orthorhombic
5	C_5.41_AF_1.72_	5.3936	5.5870	14.6245	90	440.6953	3.7800	orthorhombic
6	C_4.08_A_1.02_F	5.3704	5.5679	14.5676	90	435.5981	3.6970	orthorhombic
7	C_6.34_A_2.23_F	5.3420	5.5386	14.4977	90	428.9696	3.6140	orthorhombic
8	C_8.33_A_3.01_F	5.3155	5.4965	14.4334	90	421.6955	3.6080	orthorhombic
9	C_10.17_A_4.09_F	5.2947	5.4716	14.3918	90	416.9373	3.5910	orthorhombic

## Data Availability

Data available on request, michal.pyzalski@agh.edu.pl.
